# Fluctuating arch symmetry: a comparison of two methods of assessment - applicability and efficiency

**DOI:** 10.1590/2177-6709.29.4.e2423265.oar

**Published:** 2024-09-02

**Authors:** Maria Giulia Rezende PUCCIARELLI, Eloá Cristina Passucci AMBROSIO, Thaís Marchini OLIVEIRA, Chiarella SFORZA, Márcio de MENEZES, Simone SOARES

**Affiliations:** 1 University of São Paulo, Bauru School of Dentistry, Department of Prosthodontics and Periodontology; Hospital for Rehabilitation of Craniofacial Anomalies (HRAC/USP) (Bauru/SP, Brazil).; 2 University of São Paulo, Bauru School of Dentistry, Department of Pediatric Dentistry, Orthodontics and Public Health (Bauru/SP, Brazil).; 3 Università degli studi di Milano, Department of Biomedical Sciences for Health (Milan, Italy).; 4 Amazonas State University, School of Dentistry, Department of Operative Dentistry (Manaus/AM, Brazil).

**Keywords:** models, Asymmetry, Dental cast analysis, Dental arch, Methods, dentários, Assimetria, Análise de modelos, Arcada dentária, Métodos

## Abstract

**Introduction:**

Symmetry is balance, some correspondence in the size, form, and arrangements of parts on opposite sides of a plane, line, or point. The opposite of this concept is asymmetry, or imbalance.

**Objective:**

This retrospective study compared two methods for assessing arch symmetry with linear measurements based on triangles, to determine their applicability and efficiency.

**Methods:**

Two groups were enrolled: children (n=20) and adults (n=20), and the arch symmetry was assessed from linear measurements. Method 1: the incisor-canine (INC), canine-molar (CM), and incisor-molar (INM) distances (paired *t*-test and Pearson correlation). Method 2: a mathematical equation between the cusps measurements of the canines and the distobuccal of the first molars leading to result 1 (*t*-test for one sample and bootstrapping analysis). Dental casts were digitized and analyzed using a software program. The Bland-Altman test compared the methods (α=0.05).

**Results:**

The Bland-Altman test revealed concordance between the methods; however, separately the results were different: In method 1, the mandibular arch did not demonstrate correlation (children, INC r=0.33; CM r=0.45; INM r=0.51; adults, CM r=0.46; INM r=0.35), however, the maxilla revealed a strong correlation in children and a strong/moderate correlation in adults. In method 2, both arches were symmetrical (*p*>0.05).

**Conclusion:**

Method 1 may be appropriate during orthodontic treatment, and method 2 may be indicated for final treatment. These methods are useful; however, only method 1 identified the side of asymmetry. The methods can contribute to future studies in syndromic and non-syndromic patients, before and after orthognathic surgeries and orthodontic treatment, comparing results.

## RESUMO

**Introdução:**

Simetria é o equilíbrio ou correspondência no tamanho, na forma e na disposição das partes em lados opostos de um plano, linha ou ponto. O oposto desse conceito é assimetria ou desequilíbrio.

**Objetivo:**

Esse estudo retrospectivo comparou dois métodos de avaliação da simetria da arcada a partir de medidas lineares baseadas em triângulos, para determinar sua aplicabilidade e eficiência.

**Métodos:**

A amostra foi composta por dois grupos: crianças (n=20) e adultos (n=20), e a simetria das arcadas foi avaliada a partir de medidas lineares. Método 1: as distâncias incisivo-canino (INC), canino-molar (CM) e incisivo-molar (INM) (teste*t* pareado e correlação de Pearson). Método 2: uma equação matemática com as medidas entre as cúspides dos caninos e a as cúspides distovestibulares dos primeiros molares, levando ao resultado 1 (teste *t* para uma amostra e análise de *bootstrapping*). Os modelos dentários foram digitalizados e analisados por meio de um *software*. O teste de Bland-Altman comparou os métodos (α = 0,05).

**Resultados:**

O teste de Bland-Altman revelou concordância entre os métodos; no entanto, separadamente, os resultados foram diferentes: no Método 1, a arcada inferior não demonstrou correlação (crianças, INC r=0,33; CM r=0,45; INM r=0,51; adultos, CM r=0,46; INM r=0,35); no entanto, a arcada superior revelou uma forte correlação em crianças e uma correlação forte/moderada em adultos. No Método 2, ambas as arcadas eram simétricas (*p*>0,05).

**Conclusões:**

O Método 1 pode ser apropriado durante o tratamento ortodôntico, e o Método 2 pode ser indicado para o tratamento final. Esses métodos são úteis; entretanto, somente o Método 1 identificou o lado da assimetria. Os métodos podem contribuir para estudos futuros em pacientes sindrômicos e não sindrômicos, antes e depois de cirurgias ortognáticas e tratamento ortodôntico, comparando os resultados.

## INTRODUCTION

Symmetry represents a visual or conceptual equilibrium achieved through the proportional arrangement of parts of a whole, in contrast to the concept of asymmetry, or imbalance.^1^ ^-^ ^3^ Previous research underscored the pivotal significance of arch asymmetry, and orthodontists face challenges in diagnosing and planning the correction of this arch discrepancy.^4^ ^,^ ^5^ The etiology of asymmetry encompasses a spectrum of genetic and environmental factors, with potential skeletal, dental, or functional effects.^2^ ^,^ ^6^ External environmental factors contributing to asymmetry may include tooth extraction or trauma, further complicating diagnostic and treatment considerations.^2^

Currently, there is no consensus on the optimal methods for assessing arch symmetry, and the effectiveness of various approaches remains uncertain. Traditionally, arch symmetry has been evaluated by measuring the distance from the teeth on one side to the midline, aligned with the median palatal raphe, and comparing it with the distance from the corresponding teeth on the opposite side to the same midline.^7^ ^-^ ^11^ However, the reliability of the palatal raphe as a symmetry axis at the palate’s center has not been conclusively established.^2^ ^,^ ^4^ Some researchers have instead employed strategic dental landmarks and linear measurements based on analogous triangles to assess symmetry.^3^ ^,^ ^12^

Two methods have been described in the literature,^3^ ^,^ ^12^ that utilize dental points and linear measurements. One method, utilized in previous studies,^13^ ^,^ ^14^ segments the dental arch and evaluates the distances between specific teeth and cusps tips.^12^ This approach is well-suited for diagnosing asymmetries of skeletal, dental, or combined origin, and is essential for guiding effective treatment interventions. The other one is a geometric analysis to devise a method for assessing the symmetrical alignment of teeth within the dental arch and the overall curvature symmetry.^3^ A significant challenge lies in identifying the symmetry axis of both the upper and lower dental arches, which is compounded by the absence of specific anatomical landmarks in the central regions of the palate and mandibular body. Consequently, the principles of symmetrical figures were employed to investigate the interrelationships among the dental arch points and to derive an evaluative metric termed the IXS Index. This index yields a value of one when two pairs of dental features, such as the cusps of the canines and the mesiobuccal cusps of the first molars, are in perfectly symmetrical alignment. A deviation from 1 indicates asymmetrical positioning of the teeth.

Ensuring symmetry within individual dental arches (arch form) and establishing harmonious occlusal relationships between the maxillary and mandibular arches^15^ is crucial for managing the development of occlusion and correcting malocclusion. Evaluating arch symmetry and dimension is essential both before and after treatment in pediatric and adult populations, serving as a cornerstone for understanding malocclusion, treatment planning, and assessing treatment outcomes in orthodontics. The efficacy of the method for evaluating symmetry depends on its versatility, allowing its application across diverse demographic groups irrespective of age or inherent arch characteristics. While asymmetrical dental arches are prevalent in children^11^ due to various developmental factors, older individuals tend to exhibit arch asymmetry, due to cumulative external environmental influences over their lifetimes.^16^

This study assessed the efficacy and applicability of these two methods that utilize dental points and linear measurements, and compared their respective merits and discernment, namely which offers greater specificity and broader applicability to evaluate dental arch symmetry. The methods were evaluated using stereophotogrammetric linear measurements, offering insights into the selection of the most effective approach for potential applications in other studies. The null hypothesis tested was that no significant differences would exist between the methods for evaluating dental arch symmetry.

## MATERIAL AND METHODS

This retrospective study was approved by the ethics review board of University of São Paulo (protocols 36314820.6.0000.5441 and 48136215.0.0000.5441). The sample size was determined to detect a strong-to-moderate correlation of 0.6, with a power test of 0.8 and 0.05 significance level. The minimum number of participants per group was 20. The sample comprised 40 participants, who were separated into two groups according to age and dentition (deciduous or permanent).

### SAMPLE CHARACTERISTICS

**Figure 1: Points:**
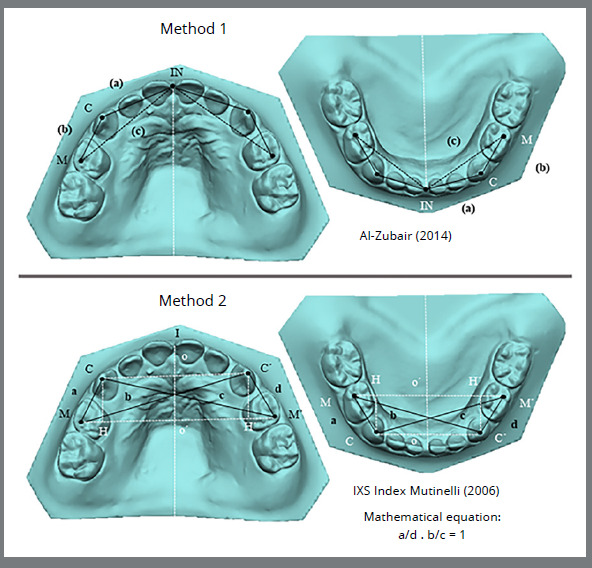
and measurements in methods of evaluating symmetry in children. IN: incisor; C: canine; M: molar. (a): from the incisal point to the canine cusp tip; (b): linear distance from the canine cusp tip to the distobuccal cusp tip of the first deciduous molar; (c): linear distance from the incisal point to the distobuccal cusp tip of the first deciduous molar (Method 1). I: incisor; C: right canine; C’: left canine; M: right molar; M’: left molar. (a): from the cusp tip of the right canine to the distobuccal cusp tip of the first deciduous right molar; (b): from the distobuccal cusp tip of the right deciduous first molar to the left canine cusp tip; (c): from the cusp tip of the right canine to the distobuccal cusp tip of the left deciduous first molar; (d): from the cusp tip of the left canine to the distobuccal cusp tip of the left deciduous first molar (Method 2).

The sample was comprised of two independent groups with different ages and characteristics. The child group comprised 20 participants aged five years, with complete deciduous dentition (10 teeth in each arch, total 20 teeth), Class I occlusal pattern, and without orthodontic intervention, caries, or severe dental crown destruction. The exclusion criteria were participants with harmful habits, the presence of syndromes and/or other anomalies, and difficult behavioral management (Fig 1).

**Figure 2: Points:**
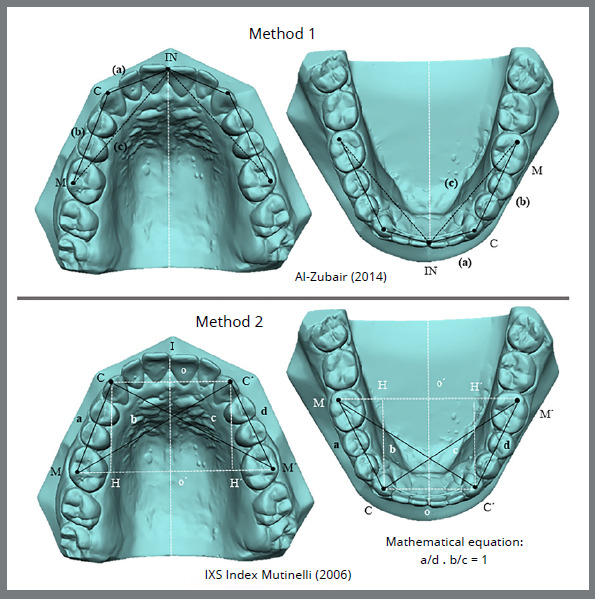
and measurements in methods for evaluating symmetry in adults. IN: incisor; C: canine; M: molar. (a): from the incisal point to the canine cusp tip; (b): linear distance from the canine cusp tip to the distobuccal cusp tip of the first permanent molar; (c): linear distance from the incisal point to the distobuccal cusp tip of the first permanent molar (Method 1). I: incisor; C: right canine; C’: left canine; M: right molar; M’: left molar. (a): from the cusp tip of the right canine to the distobuccal cusp tip of the first permanent right molar; (b): from the distobuccal cusp tip of the right permanent first molar to the left canine cusp tip; (c): from the cusp tip of the right canine to the distobuccal cusp tip of the left permanent first molar; (d): from the cusp tip of the left canine to the distobuccal cusp tip of the left permanent first molar (Method 2).

The adult group comprised 20 participants aged between 18 and 30 years, with complete permanent dentition (a total of 32 teeth with a third molar or 28 without a third molar), Angle Class III, and after orthodontic treatment with rapid maxillary expansion (RME) performed with Haas expanders attached to the canines and deciduous second molars, and a lingual bar extended to the permanent first molars. The activation protocol was the same for all participants: one full turn per day (2/4 in the morning and 2/4 in the evening) for seven days.^17^ After the active expansion, the device was maintained as retention for six months. These participants were followed-up after the skeletal maturity, with the expected orthodontic treatment completed to provide a Class I dental pattern. All adult patients were assessed after orthodontic treatment, to compare methods within the Angle Class I. Exclusion criteria were individuals with anomalies and incomplete documentation (Fig 2). 

## METHODS DESCRIPTION

### METHOD 1

#### Linear measurements

The linear measurements were as follows: the incisor-canine distance (INC), corresponding to the linear distance from the incisal point to the canine cusp tip (a); the canine-molar distance (CM), corresponding to the linear distance from the canine cusp tip to the distobuccal cusp tip of the first permanent molar (b); and the incisor-molar distance (INM), corresponding to the linear distance from the incisal point to the distobuccal cusp tip of the first permanent molar (c) as suggested by Al Zubair.^12^ These measurements were analyzed in both the child group (Fig 1) and the adult group (Fig 2).

#### Statistical analysis

The *t*-test (*t*-value) and the correlation coefficient (r-value) were applied to test the significance of differences between the right and left sides of the maxillary and mandibular segmental measurements. Statistical significance was predetermined at the 95% level, at *p* < 0.05. The outcomes were determined from tables showing the means and the standard deviations obtained using Minitab software version 19. 

### METHOD 2

#### Linear measurements

The mathematical equation for symmetry analysis can be summarized as two points being symmetrical with respect to a line if the line is perpendicular to the segment connecting the points and if it crosses the segment in the middle. Therefore, two points were identified on the curve: C symmetric to C’ and M to M’ (four points were marked). According to these criteria, the present study used: C/C´ = in the cusp tip of the canine teeth and M/ M´ = at the distobuccal cusp tip of the first molar (Figs 1 and 2). Moreover, if a line is perpendicular to two segments at the same time, the segments lie on two parallel lines: CC´ and MM´ segments are parallel to each other.^18^ Following geometric rules, these equalities were evaluated: CM = C´M´ (trapezium sides) and CM´ = C´M (trapezium diagonals). To simplify the expression, the sides and diagonals were assigned a single letter: CM = a, C´M = b, CM´ = c, and C´M´ = d. Rewriting the relationship and naming it as the IXS index, the IXS index^3^ = (a/d) · (b/c) = 1.

Therefore, if the points are symmetrical, the IXS index is 1. If the IXS index is less than 1, the trapezium is a scalene, with the two diagonals and the oblique sides of different lengths, as proposed by Mutinelli.^3^

#### Comparison of methods

Establishing a pertinent comparison between the methods for both groups require an understanding of the information provided by both methods. Both methods can be applied for digital or manual measurements, working with similar strategic points, such as canines and molars, and can be used to determine linear distances to analyze arch symmetry (Table 1). The analysis was performed with dental casts digitized by using a laser model scanner (R700^TM^; 3Shape, Copenhagen, Denmark) for all participants, and analyzed using the Vectra Analysis Module (VAM) software program (VECTRA H1; Canfield Scientific Inc., Fairfield, NJ, USA). 

[Table t1]

**Table 1: t1:** Characteristics of the two symmetry evaluation methods described in the literature.

Method 1 (Al-Zubair, 2014)	Method 2 IXS index (Mutinelli, 2006)
Three points in each arch side (left and right)	Four points
Three linear measurements in each arch side (left and right)	Four linear measurements
Correlation and comparison between left and right sides	No comparison and correlation between left and right sides
No mathematical equation	Mathematical equations
Suitable to maxillary and mandibular arches	Suitable to maxillary and mandibular arches
Detects asymmetry according to the distances	Do not detects the asymmetry distances
Three values in each arch side, according to linear measurements	A single value resulting from the mathematical symmetry equation

The first method can compare and correlate the left and right sides in the maxillary and mandibular arches.^12^ However, with the IXS index, this analysis is not possible, because the symmetry method does not divide the arches along the midline.^3^

The comparison and correlation between sides determined in Method 1 was interpreted by using the “p” and “r” values. The correlation coefficient (r-value) ranged from -1.0 to +1. When it is close to +1, this means that a relationship exists between the quantitative variables, and that they vary similarly. If the r-value is close to zero, no relationship exists between the variables. For method 2, the IXS index was applied as follows: CM/CʹMʹ = CMʹ/CʹM and (CM/CʹMʹ) x (CʹM/CMʹ) = 1. Therefore, the interpretation of the bootstrapping results was similar. If symmetry of the points was found, the outcome did not differ significantly from 1.

#### Error of method

The measurements were evaluated twice by the same examiner in 50% of the digitized models, to analyze the error of the method 15 days after the first measurement. Systematic errors were evaluated using the repeated-measures *t*-test, with random errors calculated according to Dahlberg.^19^

## RESULTS

No random or systematic errors were found (*p* ≥ 0.05).

The results were divided into the two methods, as shown in the tables and figures. In the first method,^12^ both children without orthodontic treatment and adults after orthodontic treatment had arch symmetry in the maxilla in all linear measurements, a paired *t*-test with no significant difference, and a strongly related correlation coefficient (r): for the child group, INC (r-value: 0.74), CM (r-value: 0.72), and INM (r-value: 0.85); and for the adult group, INC (r-value: 0.64), CM (r-value: 0.95), and INM (r-value: 0.86; Table 2). The opposite outcome was observed for the mandibular arch. Although the paired *t*-test did not indicate a statistically significant difference between the left and right sides, the correlation coefficient was not strongly related for most of the measures in both children and adults, except for the INC distance in the adults (r-value: 0.72; Table 2).

**Table 2:** Comparison and correlation coefficient between the left and right side of the children and adults.

Dimensions	Right side	Left side		
Children (n=20)	Mean±SD (mm)	Mean±SD (mm)	T paired value	Correlation (r-value)
Maxilla				
INC	16.36 ± 1.019	16.46 ± 0.96	0.56	0.74*
CM	9.49 ± 0.58	9.53 ± 0.73	0.76	0.72*
INM	24.35 ± 1.27	24.57 ± 1.32	0.18	0.85*
Mandible				
INC	12.44 ± 1.05	12.28 ± 0.89	0.53	0.33
CM	9.80 ± 0.78	9.76 ± 0.66	0.81	0.45
INM	20.71 ± 1.28	20.56 ± 1.24	0.60	0.51
Adults (n=20)	Mean±SD (mm)	Mean±SD (mm)	T paired value	Correlation (r-value)
Maxilla				
INC	19.52 ± 1,21	19.39 ± 1.27	0,58	0,64*
CM	27.24 ± 1.76	27.31 ± 1.81	0.60	0.95*
INM	43.04 ± 2.51	42.82 ± 2.67	0.47	0.86*
Mandible				
INC	14.33 ± 0.95	14.29 ± 0.93	0.79	0.72*
CM	25.12 ± 2.76	24.63 ± 2.53	0.43	0.46
INM	36.42 ± 2.55	35.93 ± 2.64	0.46	0.35

*Statistically significant at P<0.01. INC: Incisor-canine distance, CM: Canine-molar distance, INM: Incisor-molar distance, SD: Standard deviation.

**Figure 3:**
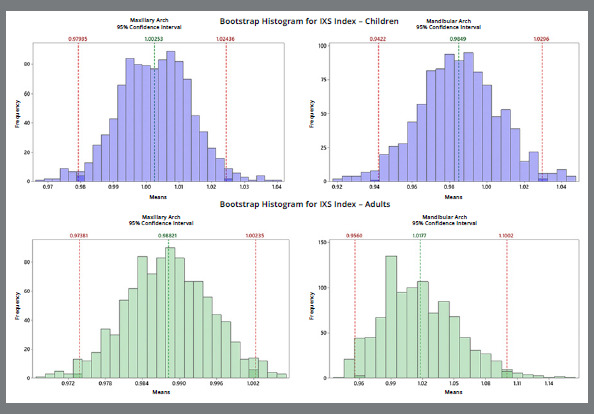
The bootstrap diagram in the maxillary and mandibular arches in children (blue) and in adults (green).

The second method evaluated was the IXS index,^3^ which is based on a mathematical equation evaluation involving the whole arch, with an IXS index of 1 indicating symmetry. The outcomes are presented in bootstrap-type resampling plots and a descriptive table. Plots in blue show evaluations of the maxillary and mandibular arch in children (Fig 3), which indicated more symmetry for the maxillary arch, with a mean of 1. However, the mandibular arch, even though it was considered symmetrical, exhibited a mean of 0.98±0.1 and variance of 0.01 (Table 3). Plots in green show evaluations of the maxillary and mandibular arch in adults (Fig 3), for which the outcome for both was arch symmetry, even though the bar graph distribution for the mandibular arch indicates more variance (value: 0.03), with a mean of 1±0.17 (Table 3).

**Table 3:** Descriptive bootstrap analysis resampling results in children and adults evaluated by IXS index.

Observed sample	Children			Adults		
	Mean ± SD	Variance	Sum	Mean ± SD	Variance	Sum
IXS INDEX Maxillary	1.00±0.05	0.002	20.05	0.988±0.0321	0.001	19.76
IXS INDEX Mandibular	0.98±0.10	0.01	19.71	1.016±0.174	0.030	20.33
Bootstrap samples for mean	Children			Adults		
	Mean ± SD	95% CI	p	Mean ± SD	95% CI	p
IXS INDEX Maxillary	1.00±0.01	0.97-1.02	0.79	0.988±0.00	0.973-1.00	0.15
IXS INDEX Mandibular	0.98±0.02	0.94-1.02	0.53	1.017±0.03	0.956-1.10	0.67

### OUTCOME COMPARISON BETWEEN THE TWO EVALUATED METHODS

The maxillary arches in both children and adults were symmetrical when using both methods. When the mandibular arch in children and adults was evaluated using Method 1, no significant correlation was found between the right and left sides, indicating a tendency toward asymmetry. However, for the children and adults evaluated using Method 2, mandibular arch symmetry was determined for both groups. 

**Figure 4:**
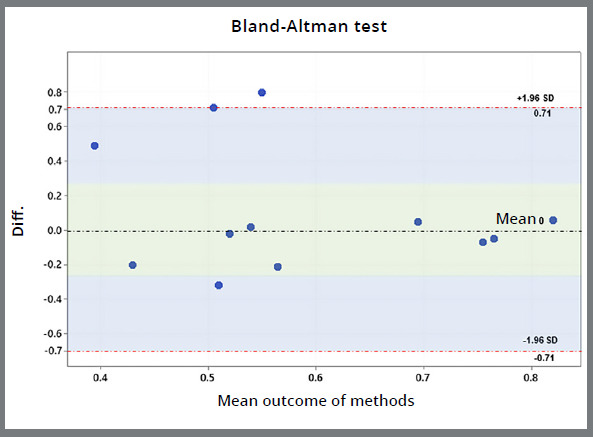
The Bland-Altman graph to compare methods.

Even though the assessment was not the same for both arches, the Bland-Altman graph showed concordance between the methods chosen, with most of the differences between the values of methods being close to zero, indicating method similarity (*p*=0.33; Fig 4). 

## DISCUSSION

The present study assessed two methods for evaluating arch symmetry through linear measurements and proposed a critical discussion of their applicability and efficiency. This information will help researchers to choose a methodology based on the answers provided by these methods. The methods were chosen based on the relevant literature, with respect to the study design and statistical analysis provided by the authors.^3^ ^,^ ^12^ The selected sample covered different phases of dental treatment and dentition (deciduous and permanent). 

The measurement methodology chosen in this study was based on stereophotogrammetry 3D analysis^20^, because it permits accurate measurements. When using calipers, it is necessary to repeat the measurements to apply the two methodologies. In stereophotogrammetry, the previously determined ratio can be used in both methods, reducing the risk of bias, and facilitating a comparison of the methods.

Previous studies have evaluated the prevalence of dentofacial skeletal mandibular asymmetry using computed tomography.^21^ ^,^ ^22^ The present study concluded that the mandibular arch tends to have more fluctuation asymmetry than does the maxillary arch, which is consistent with the results of other studies,^4^ ^,^ ^23^ although one study reported conflicting results.^24^

### CRITICAL ANALYSIS OF THE APPLICABILITY AND EFFICIENCY OF METHODS

These methods have specific particularities, with the main difference between them being whether they cross the midline or palatal raphe.^3^ ^,^ ^12^ This comparative study conclusively demonstrated that both methods are effective for different types of dentitions (deciduous and permanent) and in Angle Class III cases that become Angle Class I after treatment. It could be relevant in addressing congenital asymmetries, highlighting the importance of comparing cleft and non-cleft sides. For these patients, method 1 is more appropriate and can contribute to future studies evaluating outcomes before and after orthognathic surgery and orthodontic treatment.

Both methods evaluated in this study efficiently assessed arch symmetry. Although Method 1 seems more difficult, the answers that it can provide are more specific, and this method can identify the asymmetrical area, once the comparison and correlation have been evaluated between the right and left sides of the arch. This method may be appropriate in the planning of orthodontic treatment, because once the asymmetric region has been identified, it can be corrected with tooth alignment. Diagnosing the arch and the side on which the asymmetry is located is necessary to determine the mechanics to be applied.^25^ In this way, Method 1 can aid orthodontists in treatment planning. Method 2 could not identify the asymmetric area. The IXS index does not allow the identification of where the curve is asymmetrical on the dental arch and how much expansion or constriction is required to improve symmetry;^3^ therefore, this assessment would be appropriate as a final evaluation after orthodontics, to determine the success of the treatment. 

The differences between the methods are shown in Figure 4 (Bland-Altman). In the plot, differences close to zero demonstrate methods similarity and the widest range indicates the opposite. These divergences could be attributed to the mandibular arch because the IXS index method did not detect a fluctuating mandibular asymmetry, as in Method 1. Based on this analysis, the null hypothesis was partially rejected because the maxillae in children and adults presented symmetry, the mandible in Method 1 presented asymmetry, and the mandible in Method 2 presented symmetry.

The Bland-Altman method is usually used to compare different methodologies and was applied to the methods chosen because results close to 1 were expected, evidencing arch symmetry. Therefore, a comparison was performed by analyzing the results from the two methods (Method 1: correlation between the right and left sides, and Method 2: single-sample *t*-test). 

The anterior component of occlusal force may be related to mandibular asymmetry, originating from the mesial inclination of the teeth to the occlusal plane, and may be related to the mesial migration of the teeth and late crowding of the mandibular incisors.^26^ Other factors may include the interproximal fibers, soft tissue changes, and growth.^27^ This anterior component of force may correlate with bite strength, masticatory function, age, sex, and craniofacial pattern.^28^

Missing teeth have been reported to cause asymmetry. However, a previous study^29^ found that mandibular asymmetry in adults was not associated with the absence of teeth in the posterior region of the dental arch.

In the present study, the adult group was classified as Angle Class III malocclusion, and one study detected the highest fluctuating asymmetry in Angle Class III malocclusion. Individuals with Class III malocclusion have been identified as having the highest levels of genetic and environmental stress during their early development.^4^

### LIMITATIONS AND FUTURE PERSPECTIVES

The limitations of the present study included the groups evaluated (no craniofacial group, no mixed dentition, and no adolescents), the type of orthodontic treatment (RME, rapid maxillary expansion in the adult group), and methods used. The methodologies presented herein do not permit the diagnosis of which tooth was asymmetric. In the first method, the evaluation was based on the side (left or right) and the linear measurements. In the second method, the total arch was evaluated using a mathematical equation. This study focused on evaluating patients from Angle Class III (the most severe classification) to Class I; however, other classes and types of treatment should also be assessed. Future studies are necessary to compare other methodologies in participants of different ages and sexes or those who have received orthognathic surgery; these variables were excluded from the present study.

## CONCLUSIONS

The two methods are useful for asymmetry analysis. Method 1 can evaluates the side on which the asymmetry is present, and Method 2 provides a complete-arch evaluation, based on a mathematical equation.

## References

[1] Fischer B (1954). Asymmetries of the Dentofacial Complex. Angle Orthod.

[2] Lundström A (1961). Some asymmetries of the dental arches, jaws, and skull, and their etiological significance. Am J Orthod.

[3] Mutinelli S (2006). Symmetry evaluation in the dental arch the IXS index. Prog Orthod.

[4] Škrinjarić A, Šlaj M, Šlaj M (2018). Fluctuating dental arch asymmetry in different malocclusion groups. Acta Stomatol Croat.

[5] Veli I, Yuksel B, Uysal T (2014). Longitudinal evaluation of dental arch asymmetry in Class II subdivision malocclusion with 3-dimensional digital models. Am J Orthod Dentofacial Orthop.

[6] Bishara SE, Burkey PS, Kharouf JG (1994). Dental and facial asymmetries a review. Angle Orthod.

[7] Burstone CJ (1998). Diagnosis and treatment planning of patients with asymmetries. Semin Orthod.

[8] Kusnoto J, Evans CA, BeGole EA, Obrez A (2002). Orthodontic correction of transverse arch asymmetries. Am J Orthod Dentofacial Orthop.

[9] Langberg BJ, Arai K, Miner RM (2005). Transverse skeletal and dental asymmetry in adults with unilateral lingual posterior crossbite. Am J Orthod Dentofacial Orthop.

[10] Lear CS (1968). Symmetry analyses of the palate and maxillary dental arch. Angle Orthod.

[11] Maurice TJ, Kula K (1998). Dental arch asymmetry in the mixed dentition. Angle Orthod.

[12] Al-Zubair NM (2014). Dental arch asymmetry. Eur J Dent.

[13] Pucciarelli MGR, Toyoshima GH, Cardoso JF, Oliveira TM, Neppelenbroek KH, Soares S (2021). Arch asymmetry in patients with cleft lip and palate after rehabilitation treatment using stereophotogrammetry. J Craniofac Surg.

[14] Cardoso JF, Pucciarelli MGR, Laurenti JAS, Laposta AFE, Neppelenbroek KH, Oliveira TM (2023). Arch symmetry in patients without and with cleft lip and palate after orthodontic/rehabilitative treatment a stereophotogrammetry study. Cleft Palate Craniofac J.

[15] Kronmiller JE (1998). Development of asymmetries. Semin Orthod.

[16] Smith RJ, Bailit HL (1979). Prevalence and etiology of asymmetries in occlusion. Angle Orthod.

[17] Façanha AJ, Lara TS, Garib DG, Silva OG (2014). Transverse effect of Haas and Hyrax appliances on the upper dental arch in patients with unilateral complete cleft lip and palate a comparative study. Dental Press J Orthod.

[18] Cateni FR (1982). Il pensiero geometrico.

[19] Dahlberg G (1940). Statistical methods for medical and biological students.

[20] Rezende Pucciarelli MG, Lima Toyoshima GH, Marchini Oliveira T, Marques Honório H, Sforza C, Soares S (2020). Assessment of dental arch stability after orthodontic treatment and oral rehabilitation in complete unilateral cleft lip and palate and non-clefts patients using 3D stereophotogrammetry. BMC Oral Health.

[21] Severt TR, Proffit WR (1997). The prevalence of facial asymmetry in the dentofacial deformities population at the University of North Carolina. Int J Adult Orthodon Orthognath Surg.

[22] Willems G, De Bruyne I, Verdonck A, Fieuws S, Carels C (2001). Prevalence of dentofacial characteristics in a belgian orthodontic population. Clin Oral Investig.

[23] Scanavini PE, Paranhos LR, Torres FC, Vasconcelos MHF, Jóias RP, Scanavini MA (2012). Evaluation of the dental arch asymmetry in natural normal occlusion and Class II malocclusion individuals. Dental Press J Orthod.

[24] Le B, Nielsen B (2015). Esthetic implant site development. Oral Maxillofac Surg Clin North Am.

[25] Ruellas AC, Koerich L, Baratieri C, Mattos CT, Alves M, Brunetto D (2014). Reliability of CBCT in the diagnosis of dental asymmetry. Dental Press J Orthod.

[26] Acar A, Alcan T, Erverdi N (2002). Evaluation of the relationship between the anterior component of occlusal force and postretention crowding. Am J Orthod Dentofacial Orthop.

[27] Richardson ME (1994). The etiology of late lower arch crowding alternative to mesially directed forces a review. Am J Orthod Dentofacial Orthop.

[28] Al Qassar SS, Mavragani M, Psarras V, Halazonetis DJ (2016). The anterior component of occlusal force revisited direct measurement and theoretical considerations. Eur J Orthod.

[29] Thiesen G, Gribel BF, Pereira KC, Freitas MP (2016). Is there an association between skeletal asymmetry and tooth absence. Dental Press J Orthod.

